# A novel meniscal root refixation pull‐in technique with an all‐suture anchor shows biomechanical properties comparable to standard suture anchor and transtibial pull‐out techniques

**DOI:** 10.1002/jeo2.70310

**Published:** 2025-06-15

**Authors:** Thorben Briese, Amaris Kieninger, Christian Peez, Adrian Deichsel, Elmar Herbst, Maurice Balke, Michael J. Raschke, Christoph Kittl

**Affiliations:** ^1^ Department of Trauma, Hand‐ and Reconstructive Surgery University Hospital Münster Münster Germany; ^2^ Sportsclinic Cologne Cologne Germany

**Keywords:** all‐suture anchor meniscus root repair, meniscus root repair, meniscus root tear, transtibial pull‐out repair, suture anchor meniscus root repair

## Abstract

**Purpose:**

Repair techniques for posterior meniscal root (PMMR) tears include repair with transtibial pull‐out and anchors. An alternative approach uses all‐suture anchors pulled in, avoiding a posterior medial portal. While clinical feasibility has been assessed, biomechanical properties of this technique remain unknown. We hypothesised that the biomechanical properties using the pull‐in technique would be comparable to those achieved with conventional repair techniques.

**Methods:**

Fifty fresh‐frozen porcine tibiae were fixed in a steel pot. Whereas in group (1) the native meniscal root was kept intact (native meniscal root (NM)), the PMMR was sectioned and refixed in groups (2)–(5): (2) Double‐loaded suture anchor (screw anchor) (SA), (3) transtibial pull‐out repair with two sutures (TTPO), (4) double‐loaded pull‐in repair with all‐suture anchor that was pulled into the subcortical bone which was predrilled from retrograde direction (PULL) and (5) double‐loaded push‐in repair with all‐suture anchor traditionally pushed into the predrilled subcortical bone in antegrade direction (PUSH). Testing was performed using a universal testing machine with 1000 cycles (5–20 N/0.5 Hz) with subsequent load‐to‐failure (LTF) meaning failure of the NM or refixation. Outcomes measured included LTF (N), cyclic displacement (mm), and stiffness (N/mm). The failure mode was documented macroscopically.

**Study design:**

Controlled laboratory study.

**Results:**

No repair technique restored the stability of the NM, reaching 1064.6 ± 226.0 N in LTF (*p* ≤ 0.0001). Reconstructions had significantly lower LTF: SA (251.4 ± 52.8 N), TTPO (233.4 ± 50.0 N), PULL (206.2 ± 86.5 N) and PUSH (214.3 ± 55.2 N). The NM showed the highest stiffness with 156.1 ± 76.3 N/mm (*p* ≤ .0001) compared to (SA) 36.2 ± 10.1 N/mm, (TTPO) 33.6 ± 6.2 N/mm, (PULL) 36.8 ± 12.7 N/mm, (PUSH) 27.7 ± 6.6 N/mm. Increased displacement after 1000 cycles was shown, with (2.3 ± 0.7 mm) in PULL, only with significant differences noted between NM (1.5 ± 0.8 mm) and PUSH (3.1 ± 0.7 mm) (*p* ≤ .001), NM and SA (2.5 ± 0.8 mm) (*p* ≤ .05), and TTPO (2.1 ± 0.7 mm) and PUSH (*p* ≤ .05). No failures occurred during cyclic loading. Failure after LTF was always a suture cut‐out at the meniscus.

**Conclusion:**

Current repair techniques for posterior medial root tears do not fully restore the biomechanical properties of an intact root. The new pull‐in technique with an all‐suture anchor which is pulled in instead of pushed in shows biomechanical properties comparable to conventional methods, especially regarding LTF.

**Level of Evidence:**

There is no level of evidence as this study was an experimental laboratory study.

**Abbreviations**
ACL: anterior cruciate ligament
Fig.: figure
Hz: hertz
LTF: load‐to‐failure
mm: millimetre
MRI: magnetic resonance imaging
N: newton
NM: native meniscus
PMMR: posterior medial meniscus root
SA: suture anchor
Tab.: table
TTPO: transtibial pull‐out

## INTRODUCTION

With 10%–21% of all meniscus tears being meniscus root tears, their incidence is high [7, 23]. Whereas lateral meniscus posterior root tears often occur in anterior cruciate ligament (ACL) injuries, posterior medial meniscus root (PMMR) tears are commonly degenerative [25]. The biomechanical consequences of posterior root tears are similar to those of a complete meniscectomy [15], impairing the meniscus's important function of load‐sharing and shock absorption. If left untreated, root tears can lead to meniscal extrusion and loss of function [4, 21, 30]. Repairing the meniscal root can therefore restore normal cartilage contact pressure and reduce extrusion, which is essential for optimal clinical outcomes [15, 16, 30]. Refixation of the posterior meniscus root can be achieved through indirect fixation techniques, such as pull‐out sutures via a transtibial tunnel with extracortical fixation, or through direct fixation using suture anchors.

Previous studies have evaluated the clinical, biomechanical, and radiographic outcomes of direct arthroscopic suture anchor refixation compared to indirect pull‐out suture repairs, reporting no significant superiority of either technique [13, 14, 16]. However, the direct suture anchor techniques present challenges, as they require an additional posterior portal, which increases the risk of damaging neurovascular structures. Even with specially designed curved passing devices, secure insertion of the anchor can be difficult [3, 17]. Balke et al. developed an alternative arthroscopic technique of suture anchor repair of meniscal root tears, utilising the benefit of a subcortical fixation, without the need of an additional posterior portal [6]. This technique uses a 1.8 mm all‐suture anchor which is arthroscopically pulled into the subcortical bone of the meniscal root attachment instead of tapping it in [6]. While the clinical feasibility of this pull‐in technique has been assessed, its biomechanical properties remain unknown.

Therefore, the purpose of this study was to compare the biomechanical properties of this novel technique with standard methods. We hypothesised that the biomechanical properties of the new pull‐in technique would be comparable to those of conventional root repair techniques.

## METHODS

Ethical approval was not required for this study, as porcine knee specimens were used. A total of fifty (*n* = 50) porcine cadaveric knee specimens were included in the study. The exclusion criteria was meniscus injury. Consequently, all 50 knees could be included in the final analysis. The specimens were stored at −20°C and thawed for 24 h at room temperature before preparation and testing.

### Specimen preparation

**Figure 1:**
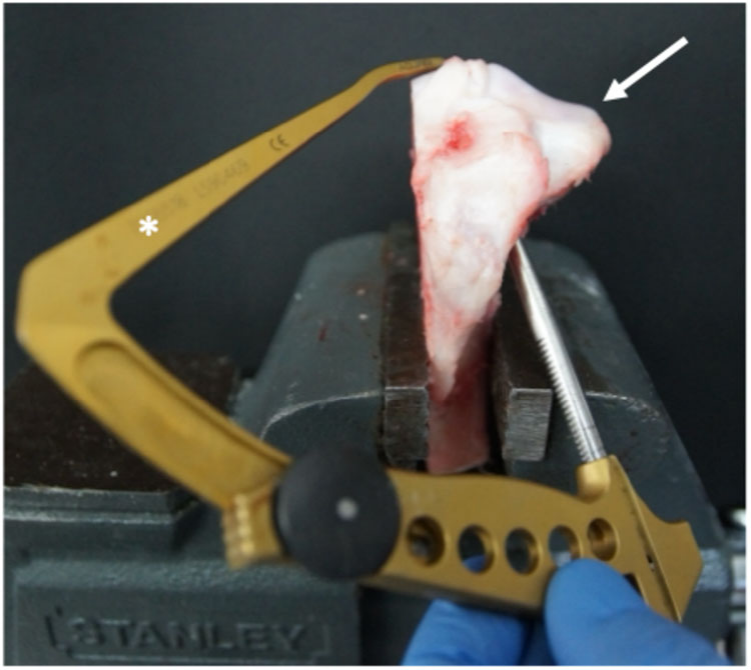
Left porcine tibia specimen (arrow) with the medial tibial plateau resected. A drilling guide (*) was used for creating a 1.8 mm drillhole at the insertion site of the posterior medial meniscus root.

**Figure 2:**
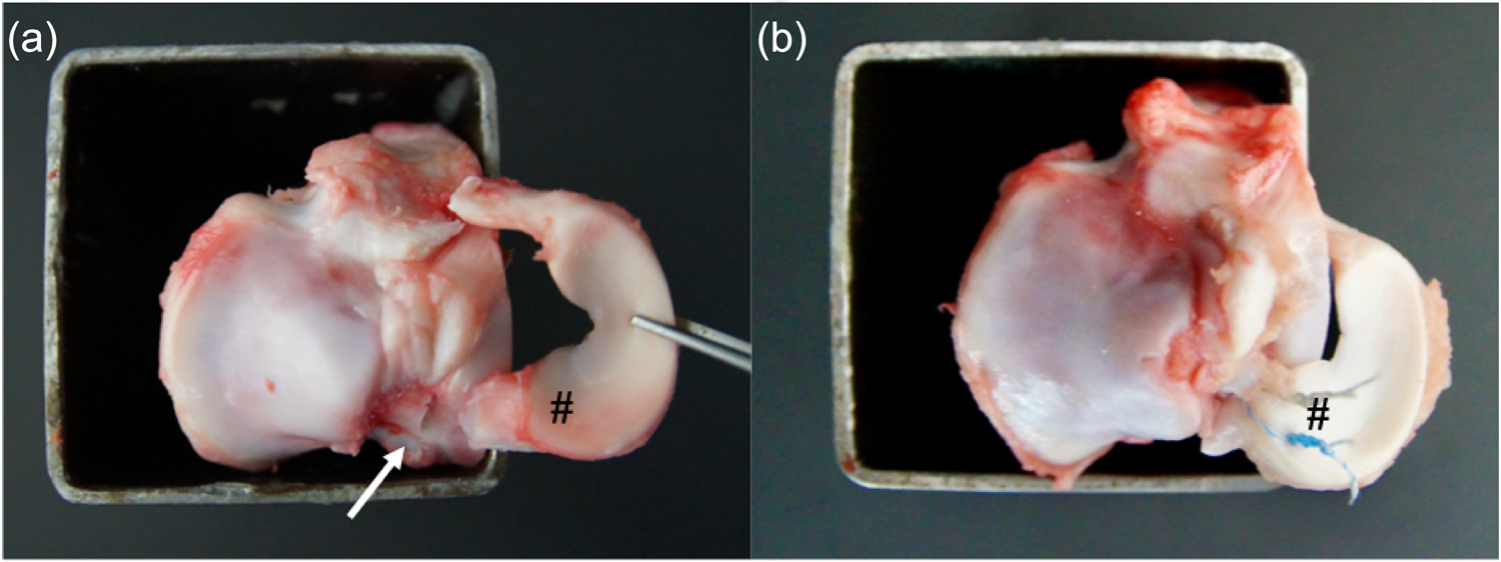
(a) Left porcine tibia specimen, without the lateral meniscus and without the cruciate ligaments. The posterior medial meniscus root (PMMR) (#) was completely dissected 5 mm from its insertion (white arrow). (b) The PMMR was refixed to its insertion with a suture anchor, and the meniscus root sutured onto the anchor with two sutures (#).

The porcine cadaveric knee specimens were stored at −20°C and thawed at room temperature for 24 h prior to preparation and testing. Preparation and testing began when the specimens reached room temperature. During dissection, the distal femur, the capsule, anterior extensor apparatus, and all associated muscles, collateral ligaments, the lateral meniscus, and cruciate ligaments were removed, leaving the proximal tibia intact while the medial meniscus remained intact at its posterior root. Then the medial tibial plateau was also resected, leaving the intercondylar region with the PMMR intact. Now the resection and refixation of the PMMR was performed according to the following protocol (Figures 1 and 2). After preparation and refixation, the porcine knee specimens were fixed in a steel pot using polymethylmethacrylate prior testing. The native porcine knee specimen was tested with an intact native meniscus root (NM) in Group 1), while for the reconstructions, the PMMR was sectioned 5 mm from its native insertion and refixed at its native insertion using various techniques (Groups (2)–(5)). Therefore, five different independent test groups were analysed (*n* = 10 each):

(1) Native meniscus root (NM).
(2) Double loaded screw anchor repair (SA).
(3) Transtibial pull‐out repair with two sutures (TTPO).
(4) Double loaded pull‐in repair (PULL).
(5) Double loaded push‐in repair (PUSH).

#### SA

The screw anchor (Super Revo 5 mm, Conmed, USA) was self‐drilled and applied directly at the insertion of the PMMR.

#### TTPO

For the TTPO technique, a 2.4‐mm K‐wire was drilled transtibial retrograde using a conventional root repair aiming device (Figure 1), targeting the PMMR insertion. Drilling was initiated from the lateral side, as the medial tibia had been resected for the test setup (Figure 1). The PMMR was sutured using two vertical sutures (#2 Hi‐Fi, Conmed, USA). These sutures were then passed through the tibial tunnel created by the K‐wire and fixed extracortical onto an endobutton, (Flipptack, Karl Storz, Germany) using a Samsung Medical Center knot, and three half‐hitches [18].

#### PULL

**Figure 3:**
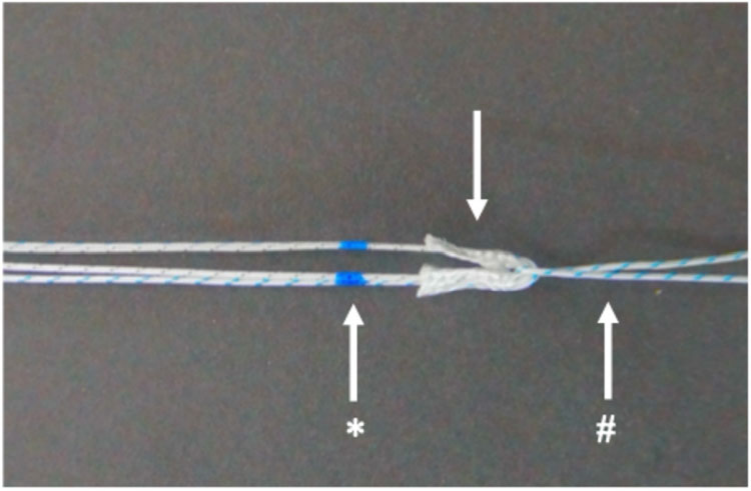
According to the technique of Balke et al. [6] a pulling suture (# = #2 Hi‐Fi suture) was looped around the suture anchor (arrow = with two #2 Hi‐Fi sutures double loaded Y‐Knot Flex 1.8 mm all‐suture anchor) that was separated from its insertion device. The necessary insertion depth was marked (*).

For the PULL technique, drilling was conducted using a 1.8‐mm disposable drill bit from Conmed using a conventional root repair device (Figure 1), targeting the PMMR insertion analog to the TTPO technique in retrograde direction. Following the method described by Balke et al. [6], a modified double‐loaded all‐suture anchor (Y‐Knot Flex 1.8 mm, Conmed, USA) (Figure 3) was then pulled into the subcortical bone using a 2# Hi‐Fi suture until the required insertion depth was achieved. The sutures of the anchor were then tensioned to deploy the anchor subcortically.

#### PUSH

In the push‐in technique, antegrade predrilling directly at the PMMR insertion was performed using a 1.8 mm disposable drill bit from Conmed, after which the anchor (Y‐Knot Flex 1.8 mm, Conmed, USA) was antegrade impacted directly at the PMMR insertion and deployed subcortically.

In all groups, one suture was positioned adjacent to the meniscus synovial junction, located 5 mm medial to the lateral edge of the posterior horn, while the second suture was placed 5 mm more anterior than the first [19] (Figure 2). In all anchor groups, the PMMR was secured to the anchor using two single stitches, followed by a Samsung Medical Center knot and three half hitches [18].

### Biomechanical testing

**Figure 4:**
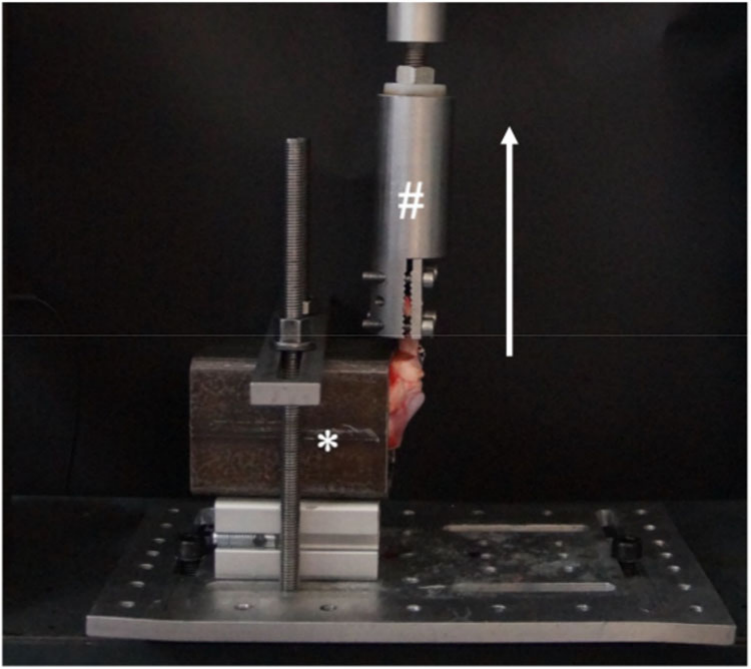
Biomechanical testing set up. The steel pot (*) is fixed to the base of the testing machine perpendicular to the clamp (#). The meniscus is then pulled in line to the original insertion of the meniscus root (see white arrow indicating the force direction of the tensile testing).

Biomechanical testing was conducted using a universal uniaxial testing machine (Zwick, Roell, Germany). The steel pot containing the embedded porcine tibia was aligned perpendicular to the testing machine. The meniscus was secured to the machine with a steel clamp, leaving a 10 mm gap between the clamp and the meniscal root insertion site as well as the sutures (Figure 4). Regarding tensile testing, a preload of 2 N was applied, followed by cyclic loading comprising 1000 cycles at a frequency of 0.5 Hz, with loads ranging from 5 to 20 N. This was succeeded by a load‐to‐failure (LTF) test, where the construct was subjected to continuous loading at a rate of 0.5 mm/s until failure occurred. The specimen was kept moisturised with water during testing. Measurements of LTF (N), displacement (mm), and stiffness (N/mm) were recorded. This testing protocol was adapted from previous biomechanical studies on meniscal root repair [13, 14]. The displacement (mm) was measured indirectly via the crossbar of the universal uniaxial material testing system. Stiffness was calculated from the slope of the linear portion of the load displacement curve during LTF. The defined mode of failure (pullout of suture or anchor or cut out of the sutures) was macroscopically documented.

### Statistical analysis and sample size calculation

Data analysis was automated using a custom‐made MATLAB programme (MATLAB, version R2020a, MathWorks). Statistical analysis was performed with PRISM (version 10, GraphPad Software). A two‐way analysis of variance (ANOVA) repeated measurement with Tukey's post hoc correction was performed for displacement and a one‐way ANOVA repeated measurement with Tukey's post hoc correction was performed for stiffness and LTF. A *p*‐value less than 0.05 was deemed to identify significant differences.

An a‐priori power analysis was performed using G*Power‐2 software (University Düsseldorf, Düsseldorf, Germany) [12]. A two‐tailed t‐test with means (difference between two independent means, two groups) was selected in G*Power‐2 for the power analysis. Based on means and standard deviations from prior studies of meniscus refixation methods in porcine knee models [13, 14] it was assumed that a sample size of 10 would allow for the identification of differences (effect size/Cohen's *d* = 2), with 80% power, at the significance level of *p* < 0.05.

## RESULTS

### LTF testing

**Figure 5:**
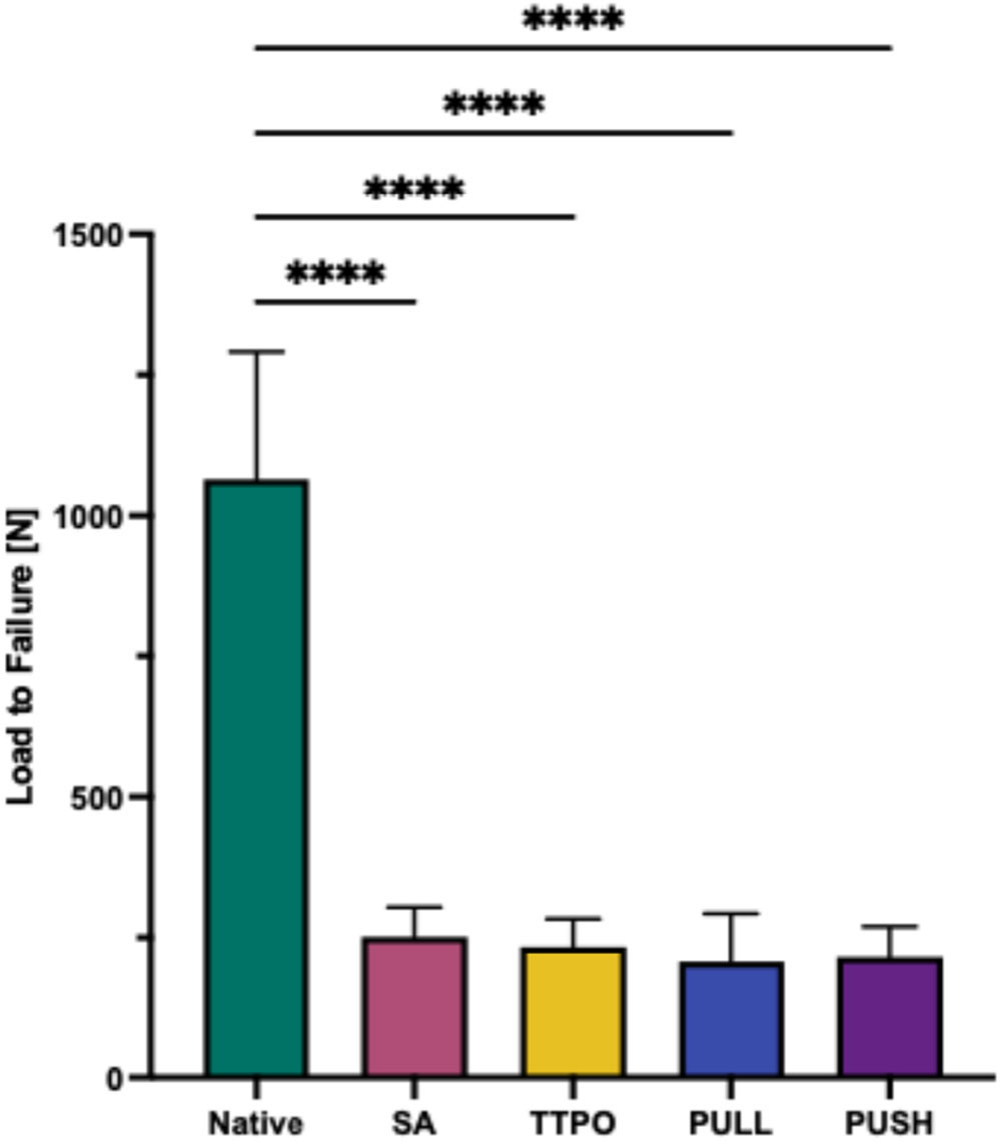
Load to failure in N of the different repairs and the native root. PULL, new pull‐in technique; PUSH, push‐in technique; native, native root; SA, screw anchor; TTPO, transtibial pull‐out. Significance was indicated by asterisks (****p ≤ 0.0001).

LTF was significantly higher (*p* ≤ 0.0001) for the NM (1064.6 ± 226.0 N) compared to the repair techniques (251.4 ± 52.8 for SA, 233.4 ± 50.0 mm for TTPO, 206.2 ± 86.5 mm for the pull‐in group, and 214.3 ± 55.2 mm for the push‐in group). There was no statistically significant difference between the repair techniques (Figure 5).

### Stiffness

**Figure 6:**
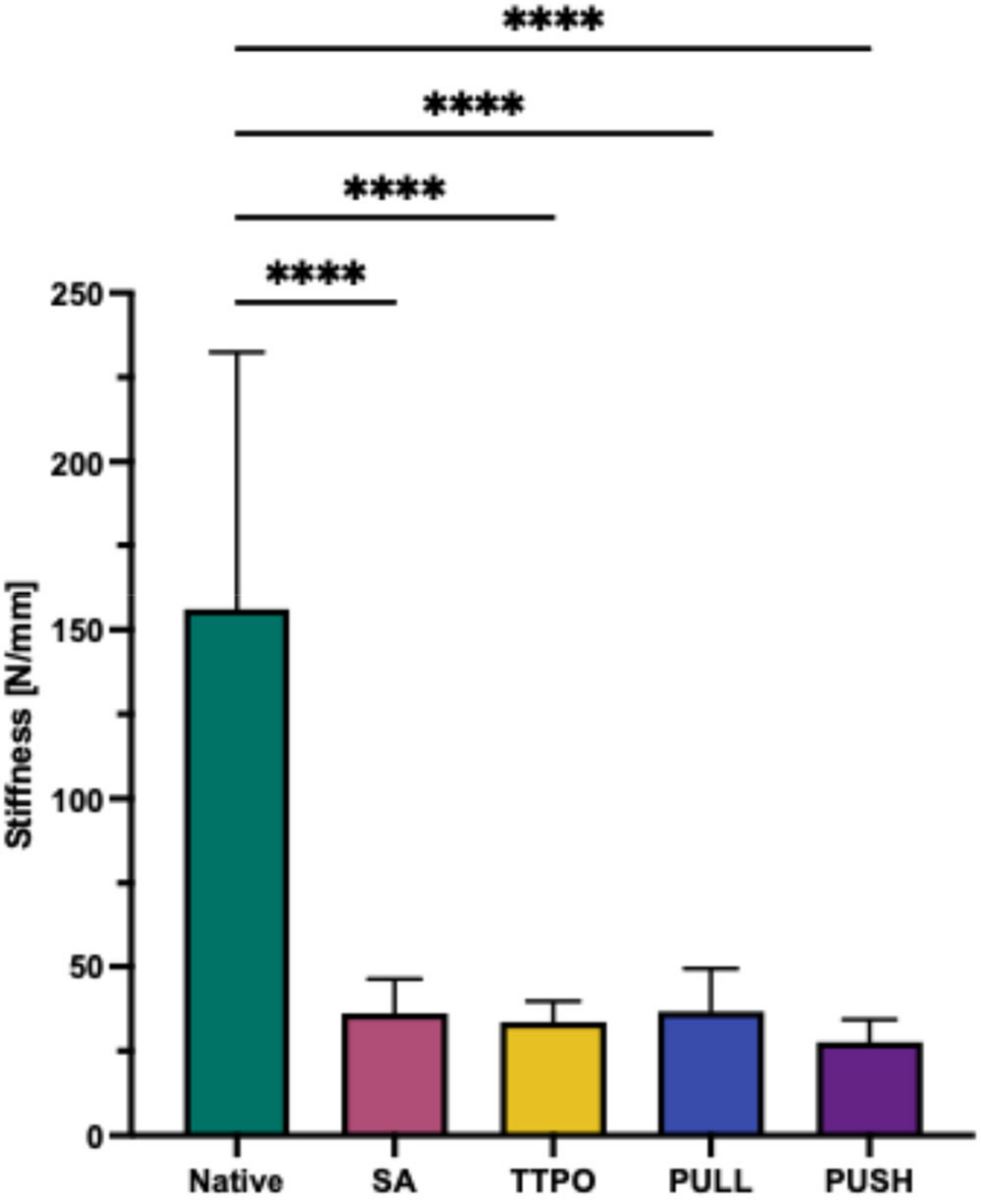
Stiffness of the different repairs and the native root (native) in N/mm. native: native root; PULL, new pull‐in technique; PUSH, push‐in technique; SA, screw anchor; TTPO, transtibial pull‐out. Significance was indicated by asterisks (****p ≤ 0.0001).

The NM showed the highest stiffness 156.1 ± 76.3 N/mm (*p* ≤ 0.0001) compared to the different repair techniques, which was 36.2 ± 10.1 N/mm for the SA group, 33.6 ± 6.2 N/mm for the TTPO group, 36.8 ± 12.7 N/mm in the pull‐in group, and 27.7 ± 6.6 N/mm in the push‐in group (Figure 6). There was no statistically significant difference between the repair techniques.

### Displacement during cyclic loading

**Figure 7:**
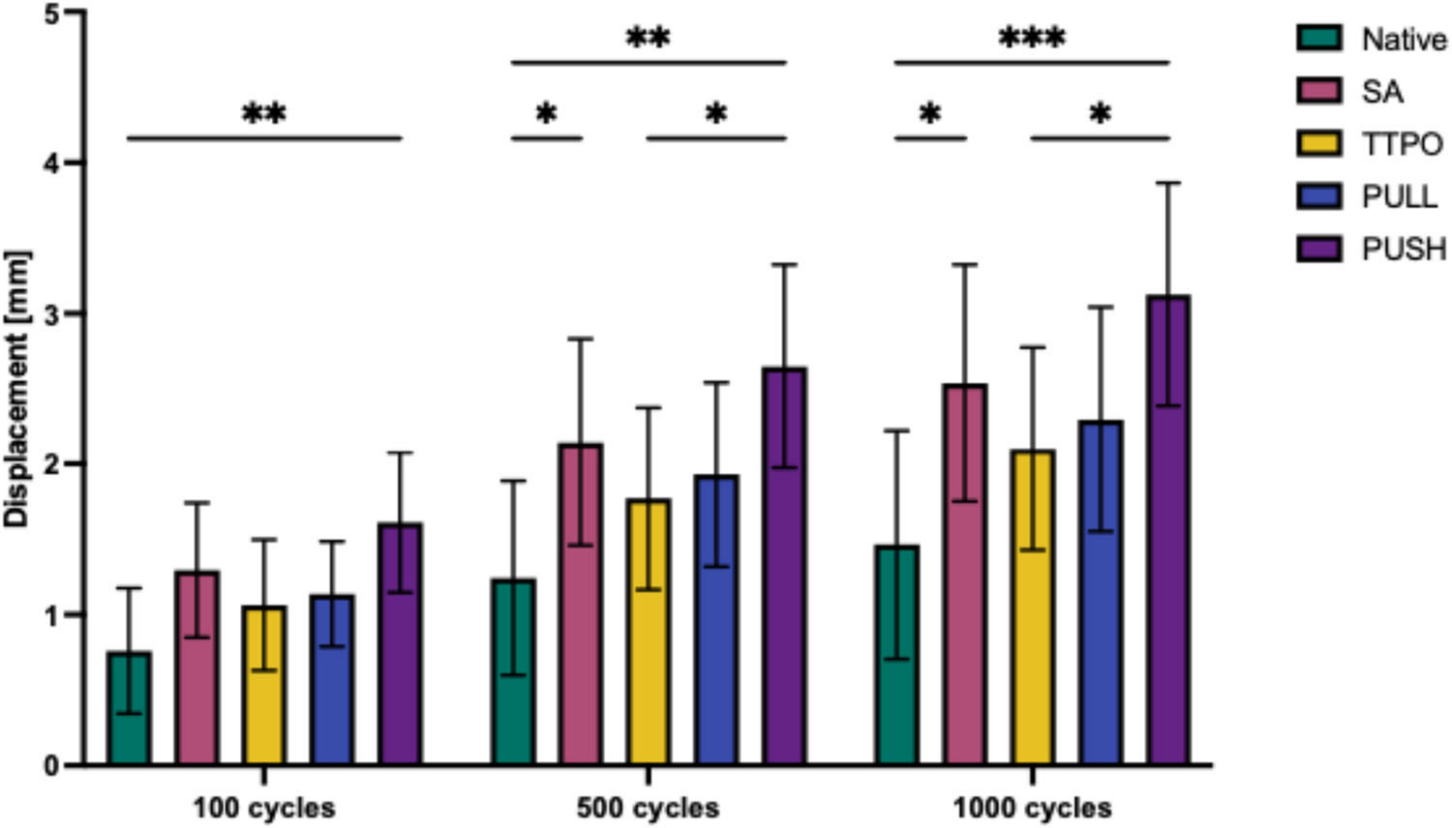
Displacement of the different repairs and the native root at 100, 500 and 1000 cycles. native, native root; PULL, new pull‐in technique; PUSH, push‐in technique; SA, screw anchor; TTPO, transtibial pull‐out. Displacement is presented in mm; Significance was indicated by asterisks (ns: not significant; *p ≤ 0.05; **p ≤ 0.01; ***p ≤ 0.001).

Displacement during cyclic loading was recorded after 100, 500, and 1000 cycles (Table 1 and Figure 7). All reconstructions as well as the NM showed an increase of the displacement during cyclic loading. After 100 cycles, a significant difference was only observed between the NM and the PUSH refixation (*p* ≤ 0.01). After 500 cycles, significant differences were observed between NM and PUSH (*p* ≤ 0.01), between NM and SA (*p* ≤ 0.05), and TTPO and PUSH (*p* ≤ 0.05). After 1000 cycles, significant differences were observed between NM and PUSH (*p* ≤ 0.001), between NM and SA (*p* ≤ 0.05) and TTPO and PUSH (*p* ≤ 0.05). No significant differences were observed between SA, TTPO and the new PULL technique.

**Table 1:** Displacement during cyclic loading of the different repairs.

Displacement during cyclic loading in mm±SD			
Cycles	100	500	1000
Native	0.8 ± 0.4	1.2 ± 0.6	1.5 ± 0.8
SA	1.3 ± 0.4	2.2 ± 0.7*	2.5 ± 0.8*
TTPO	1.1 ± 0.4	1.8 ± 0.6	2.1 ± 0.7
PULL	1.1 ± 0.3	1.9 ± 0.6	2.3 ± 0.7
PUSH	1.6 ± 0.5*	2.7 ± 0.7*	3.1 ± 0.7*

*Note*: Data is presented in mm±SD. Significant changes compared to the native root are indicated with *.

Abbreviations: PULL, new pull‐in technique; PUSH, push‐in technique; SA, screw anchor; TTPO, transtibial pull‐out.

### Failure mode

None of the reconstructions failed during cyclic loading. All failures occurred during final load to failure. The failure mode in all reconstructions was always a cut out of the sutures in the meniscus. No anchor failure itself was observed in all groups, nor any failure at the knot itself. The native meniscus always failed as an avulsion at its tibial origin.

## DISCUSSION

The purpose of this study was to compare the biomechanical properties of all‐suture anchors using both push‐in and pull‐in techniques against screw anchors and TTPO repairs. The key finding, consistent with our hypothesis, is that none of the tested repair techniques restored the structural properties of the intact posterior meniscus root concerning load to failure, displacement, and stiffness. Furthermore, all refixation techniques demonstrated comparable biomechanical properties. Notably, failure of meniscal refixation during cyclic loading did not occur with any of the tested methods. During the LTF testing, the mode of failure consistently involved the sutures cutting out of the meniscus, while the anchors or extracortical buttons remained stable and did not displace. Our data also supports the biomechanical feasibility of the new PULL technique using all‐suture anchors for PMMR repair. Thus, based on our findings, all tested refixation techniques appear suitable for clinical practice, depending on the surgeon's preference.

In previous studies, the biomechanical properties of suture anchor refixation have been investigated, showing promising results [13, 14]. However, the biomechanical properties of all‐suture anchor refixation for meniscus root tears remain largely unexplored. Contrary to our results, Feucht et al. [13] reported that suture anchors were superior to TTPO techniques in terms of cyclic displacement, with displacements of 0.6 mm versus 1.0 mm after 100 cycles, 1.0 mm versus 1.8 mm after 500 cycles, and 1.3 mm versus 2.2 mm after 1000 cycles, respectively. In our study, TTPO demonstrated a non‐significant difference compared to screw anchors and all‐suture anchors concerning cyclic displacement. Feucht et al. [13], however, employed a different suture anchor (5.5 mm Corkscrew FTII, Arthrex) and a different extracortical fixation device (Button Plate, Synthes), although they utilised similar suture techniques. Forkel et al. [14] reported similar findings, using a combined ACL TTPO tunnel for their pull‐out sutures. Discussing if one or two tunnel techniques in TTPO should be applied, La Prade et al. [22] already demonstrated no significant difference concerning primary biomechanical stability between one‐tunnel and two‐tunnel techniques. Concerning stiffness, Feucht et al. [13] showed 28.4 N/mm for the SA and 23.7 N/mm for TTPO, which is similar to our results which was 36.2 N/mm for the SA group and 33.6 N/mm for the TTPO group. Concerning LTF Feucht et al. [13] showed 241.0 N for SA and 180.1 N for TTPO which was a greater difference as in our study with 251.4 N for SA and 233.4 N for TTPO, whereas we also showed no significant difference. Notably, the all‐suture anchor exhibited no significant difference when pulled (PULL) into the porcine PMMR or being tapped (PUSH) in. Furthermore, it has to be mentioned that the biomechanical performance of all‐suture anchors is depending on its size as shown by Alt et al. [5], whereas they tested 1.6, 1.9 and 2.6 mm all‐suture anchors. Therefore, using all‐suture anchors with a greater diameter for PMMR repair might increase primary biomechanical stability.

The clinical relevance of this study lies in demonstrating the biomechanical feasibility of using all‐suture anchors with a new pull‐in technique, as opposed to a tapped‐in approach, for PMMR repairs. Additionally, our findings indicate that there is no significant clinical difference in the choice of PMMR repair technique. This is supported by previous clinical studies that analysed the clinical and radiographic outcomes of suture anchor repairs compared to pull‐out suture repairs for PMMR tears, both of which demonstrated significant functional improvements [16], whereas suture anchor repair showed slight advantages concerning radiographic (MRI) outcome. An important advantage of the pull‐in technique is the elimination of the need for an additional posterior portal if refixation with an anchor is intended, which facilitates a minimally invasive suture anchor refixation while still utilising all‐suture anchors for PMMR repair [6]. As other specific designed all‐inside all‐suture anchors for PMMR repair are designed and clinically applied [24, 32], yet their biomechanical properties remain unknown. Since all tested groups exhibited suture cutout from the meniscus as the mode of failure, using tapes may reduce the risk of failure. Previous studies have demonstrated that TTPO repairs utilising tape achieve superior maximum failure loads compared to standard No. 2 sutures [27, 29]. Additionally, Camarda et al. [10] demonstrated that using three single loops for PMMR repair enhances stability. However, given the variety of suture techniques and materials described for root repair [2, 11, 13, 14, 20, 26, 29], our study only focused on comparing TTPO and suture anchor techniques using a consistent suturing method across all specimens. Regarding in vivo loads on the PMMR, Starke et al. reported that forces on repaired menisci can reach up to 60.1 ± 20.2 N under a 500 N load in 90° flexion [31]. In our study, all refixation techniques exceeded 60 N, with LTF values ranging from 206.2 to 251.4 N. Therefore, according to our results, we suggest that all the tested techniques may be suitable for PMMR repair. As the pull‐in and the TTPO technique do not require a posterior portal, their application seems favourable, even being able to perform an all‐suture anchor refixation with the new pull‐in technique.

This study had several limitations inherent to ex vivo biomechanical studies that must be considered before transferring our results into clinical practice. Although previous studies have shown suitability of the porcine knee for use in biomechanical investigations [28], bone density in the porcine model is higher in comparison with the human knee joint [1]. The biomechanical stability of anchor refixation is assumed to depend on bone density, therefore, pullout of the anchors may occur with a higher incidence in lower bone density. Furthermore, biomechanical testing was a simulation of forces acting at time zero, and biological factors and healing of the PMMR were not considered. Nevertheless, the porcine model has already been evaluated and proven in previous biomechanical studies investigating suture anchor repairs [8, 9, 13, 14]. Additionally, various other implants and sutures are available that may exhibit different ultimate strength and stiffness values. Our study evaluated only one representative of each technique, which limited our ability to assess a broader range of implants. A further limitation is the used surgical knot for all suture anchors (Samsung Medical Center knot with three half hitches [18]) which is a very complex an stable knot that might not always be applied in clinical practice. Nevertheless, this knot was adapted from a previous biomechanical study on suture anchor refixation of PMMR [13] providing a comparable fixation in the present study. Another limitation stems from our use of lateral drilling, which could impact the pull‐out technique due to variations in the angles of refixation, potentially altering the way sutures interact with the subchondral bone. Additionally, a further limitation is that we indirectly recorded the displacement from the universal testing machine, meaning we were not able to record the direct derivation of the actual displacement of the PMMR.

## CONCLUSION

Current repair techniques for posterior medial root tears do not fully restore the biomechanical properties of an intact root. The new pull‐in technique with an all‐suture anchor which is pulled in instead of pushed in shows biomechanical properties comparable to conventional methods, especially regarding LTF.

## AUTHOR CONTRIBUTIONS

**Thorben Briese**: Conception and design; testing and data acquisition; statistical analysis; writing. **Amaris Kieninger**: Testing and data acquisition; statistical analysis. **Christian Peez**: Internal review; statistical analysis. **Adrian Deichsel**: Internal review. **Elmar Herbst**: Internal review. **Maurice Balke**: Conception; internal review. **Michael J. Raschke**: Internal review. **Christoph Kittl**: Conception and design; testing and data acquisition; statistical analysis; writing.

## CONFLICT OF INTEREST STATEMENT

Elmar Herbst is Deputy Editor‐in‐Chief for the Knee Surgery, Sports Traumatology and Arthroscopy (KSSTA). Adrian Deichsel is Web Editor for the Knee Surgery, Sports Traumatology and Arthroscopy (KSSTA). All other authors declare no conflict of interest.

## ETHICS STATEMENT

Ethical approval from the Institutional Review Board of the University of Münster was not required for this study, as porcine specimens were used.

## Data Availability

Data are available from the corresponding author upon reasonable request.
